# Comparing the effects of focal and conventional tDCS on motor skill learning: A proof of principle study

**DOI:** 10.1016/j.neures.2022.01.006

**Published:** 2022-05

**Authors:** Aline Iannone, Irene Santiago, Silvia T. Ajao, Joaquim Brasil-Neto, John C. Rothwell, Danny A. Spampinato

**Affiliations:** a Department of Clinical and Movement Neurosciences, University College London, London, UK; b Department of Physiological Sciences, Biology Institute, University of Brasília, Brasília, DF, Brazil; c Department of Biomedicine, University of Sevilla, Sevilla, Spain; d Department of Psychological Sciences, University of Liverpool, Liverpool, UK; e Department of Clinical and Behavioural Neurology, IRCCS Santa Lucia Foundation, Rome, Italy

**Keywords:** TMS, tDCS, Motor learning, Motor cortex

## Abstract

Transcranial direct current stimulation (tDCS) has emerged as a promising intervention in clinical and behavioral neuroscience; however, the response variability to this technique has limited its impact, partly due to the widespread of current flow with conventional methods. Here, we investigate whether a more targeted, focal approach over the primary motor cortex (M1) is advantageous for motor learning and targeting specific neuronal populations. Our preliminary results show that focal stimulation leads to enhanced skill learning and differentially recruits distinct pathways to M1. This finding suggests that focal tDCS approaches may improve the outcomes of future studies aiming to enhance behavior.

## Highlights

• We compare the after-effects of conventional TDCS to a more focal, targeted approach.
• TDCS montage selection influences motor skill learning.
• We show that it also influences neurophysiological outcomes measured with directional TMS.

Previous neurophysiological work has shown that outcomes of transcranial direct current stimulation (TDCS) are influenced by the current flow between the electrodes (Rawji et al., 2018). When targeting the motor cortex (M1), conventional tDCS montage calls for a large (e.g., 5 × 5 cm) anode electrode to be positioned over the M1 hotspot of the right hand, while a large cathode electrode is placed over the right supra-orbital area (Woods et al., 2016). Although this setup can modulate transcranial magnetic stimulation (TMS) triggered motor-evoked potentials (MEPs) and enhance motor learning (Reis et al., 2009), modeling work has demonstrated that current flow distribution is widespread and not directly underneath the electrodes (Datta et al., 2009). As a result, the current flow of tDCS passes through both structurally and functionally distinct brain areas, which likely contributes to the well-known variability of tDCS-induced effects across physiological and behavioral studies (Wiethoff et al., 2014).

To combat this issue, a more focal montage has been introduced to restrict the current flow by placing small electrodes (3.14 cm^2^) in a 4 × 1 ring configuration, separated by 3.5 cm, and centered over M1. While this focal montage can induce greater changes in cortical excitability and facilitate movement response times when compared to the conventional montage (Masina et al., 2021; Kuo et al., 2013), it remains unclear whether a more-targeted approach is beneficial for motor learning. Indeed, utilizing the 4 × 1 ring configuration in the context of motor learning has led to some mixed results (Doppelmayr et al., 2016; Cole et al., 2018; Ballard et al., 2021), which likely depend on the target-site (Lefebvre et al., 2019) and stimulation intensity (Lerner et al., 2021). Of these studies, only Cole and colleagues have compared the effects of focal and conventional tDCS on fine motor skill performance in children and found that both montages similarly improved manual dexterity compared to sham.

Here, we investigated whether tDCS focality plays a significant role in enhancing the learning of a de-novo motor skill in healthy young adults measured across multiple days. Since M1 is viewed as a central region involved in the encoding of recently learned movements (for review, Spampinato and Celnik, 2021), we hypothesized that the focal tDCS would produce more prominent effects on motor skill retention (measured 24 h after training) when compared to conventional and sham stimulation.

**Fig. 1:**
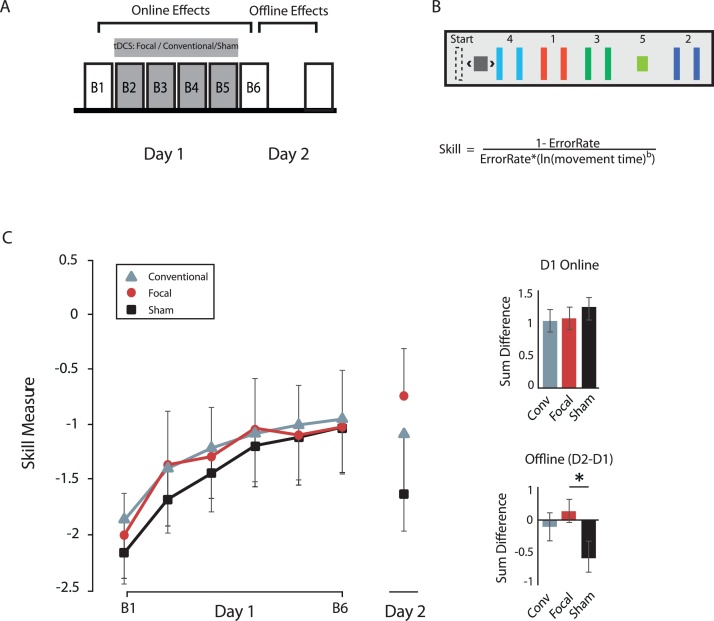
(A) Experimental Design for all groups. Individuals participated in a multiday motor-skill training, in which participants received 2 mA of either conventional, focal, or sham tDCS over M1 during training (Blocks 2-5, grey blocks). (B) Participants performed the sequential-visual-isometric-pinch task (SVIPT), which requires moving an on-screen cursor (black square) between the “Start” position and five targets by pressing down on a force transducer. Participants were instructed to improve both their speed and accuracy throughout training. The *Skill* equation has been previously used to estimate changes in the speed-accuracy trade-off, where b = 5.424 and includes the average error-rate and movement duration of each block. (C) Skill performances are plotted for Conventional (triangle), Focal (circle) and Sham (square) groups. The y-axis depicts the Skill score and the x-axis the training block. Bar graphs show group averages for the sum of on-line and off-line changes. *p < 0.05 (with Bonferroni’s multiple comparison).

We recruited 30 right-handed participants (17 females; mean age: 24.2 ± 0.92 years) who reported no contraindications to brain stimulation. Participants were randomly assigned to one of three groups: Conventional (n = 10; 6 females; mean age: 24.3 ± 1.41 years) Focal (n = 10; 5 females; mean age: 24.5 ± 1.71 years) or Sham (n = 10; 6 females; mean age: 23.7 ± 1.52 years). On the first experimental session, all individuals completed 180 trials (i.e., 6 blocks) of the sequential-visuomotor-isometric-pinch-force-task for approximately 40 min (SVIPT; Fig. 1A). Conventional, Focal or Sham tDCS was applied over the M1 hotspot during training blocks 2–5 (stimulation duration ∼ 25 min), with a current intensity set to 2 mA. Stimulation was ramped up for a 30 s period at the onset of stimulation and was maintained until participants completed block 5 of the SVIPT. After completion, stimulation ramped down for 30 s. SVIPT Training Blocks 1 and 6 were always performed without stimulation. On the following day, all individuals completed a post-test of 30 trials to assess 24 -h skill retention. In the final experimental session (separated by at least 48 h following the motor retention session), we measured the effects of tDCS montage on cortical excitability from different TMS currents over M1.

Conventional tDCS was delivered through two 25 cm^2^ sponge electrodes soaked in a saline solution (neuroConn, Munich, Germany). In this montage, the anode electrode was placed over the contralateral M1 corticomotor representation of the right FDI muscle and the cathode over the ipsilateral supra-orbital area. On the other hand, Focal tDCS was delivered by the Starstim system (Neuroelectrics, Barcelona, Spain) by using an anode 4 × 1 high-definition tDCS ring montage (Kuo et al., 2013). In this montage, the anode center electrode is placed over the M1 representational area of the right FDI muscle and is surrounded by four cathode disc electrodes (3.14 cm^2^) in order to help focalize the area of stimulation. The radii of the cathode return electrodes to the anode was set at a center-to-center distance of 3.5 cm. Sham tDCS was applied with a ramp-up 30 s/ramp-down 30 s of 2 mA tDCS for blinding purposes and individuals in this group were either administered with conventional or focal settings (5 individuals per montage). Their selected montage was maintained for all experimental sessions (e.g. behavioral and physiological assessment). Two experimenters were present at the time of stimulation in which one delivered the intervention (real or sham), while the other (blinded to the type of stimulation) ran the experiment. Thus, both the experimenter and participant were unaware of the stimulation condition. Finally, a third individual unaware of the group assignment and who did not participate in the data collection experiment was responsible for analyzing all behavioral and physiological data.

To perform the SVIPT, participants were instructed to press down on a force-transducer using their index finger, thereby controlling the movement of an on-screen cursor (Fig. 1B). The overall goal was to move the cursor between “HOME” and five targets as quickly and accurately as possible. Movement time was measured as the total time needed to reach all five targets. A trial was only considered correct if participants landed within each target. To quantify SVIPT learning, we determined changes in the speed-accuracy tradeoff function (SAF) (see equation, Fig. 1B), as previously described (Reis et al., 2009; Cantarero et al., 2015). Improvements in one parameter, without the expense of the other, can shift the overall speed-accuracy tradeoff function, reflected by an increased skill measure. On-line gains were measured as the sum of the difference between Blocks 1 and 6 of day 1. Off-line effects were calculated as the sum of differences between the first block of day 2 minus the last block of day 1.

**Fig. 2:**
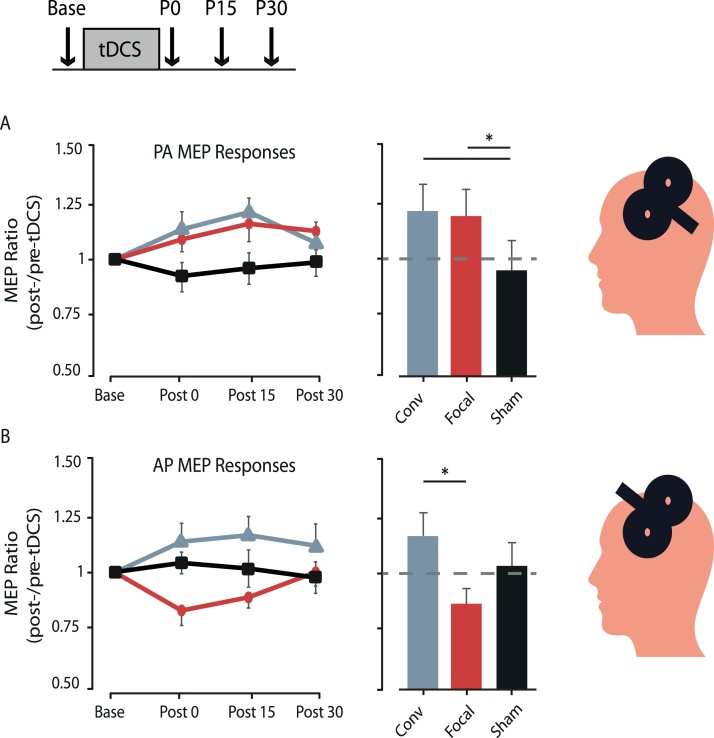
Effect of tDCS montage on the MEP amplitude evoked by (A) PA-TMS and (B) AP-TMS. y-Axis shows the mean MEP amplitude normalized to baseline measures, and the x-axis represents the MEP measurement recorded before (Base) and after tDCS (Post 0, 15, 30). Responses to conventional, focal and sham stimulation are plotted . Bar plots represent the overall mean (±SEM) post-stimulation effects (averaged from P0 to P30). Asterisks represent significant differences (*p* < 0.05 with Bonferroni's multiple corrections).

In a separate experimental session, where participants did not perform on the SVIPT, we assessed how each montage affected distinct inputs to the cortical-spinal tract (Fig. 2). To do this, TMS was applied with a figure-of-eight coil (Magstim Company Ltd, UK) over the M1 representation of the right first dorsal interosseous (FDI) muscle. MEPs were measured with electromyography. Two current directions were applied throughout the stimulation-only session: posterior-to-anterior (PA) and anterior-to-posterior (AP). MEPs were evoked using these current directions since recent work has argued PA- and AP-TMS activates separate interneuron networks that play distinct roles in physiological plasticity and motor learning (Hamada et al., 2014). The rest motor threshold (RMT), the minimum intensity needed to elicit 50 u V MEPs on 5-of-10 pulses, was determined for each direction (Rossi et al., 2009). The TMS intensity used for each current direction was set to 120 % RMT, in which 15 MEPs with PA currents and 15 MEPs with AP currents were recorded at each stimulation time point. To assess the potentiation effects of tDCS, we collected PA- and AP-MEPs prior to and after administering 20 min of 2 mA tDCS, with post-tDCS MEPs collected every 15 min up to 30 min (i.e. Post 0, Post 15, Post 30). Sham stimulation in these sessions consisted of 30 s of ramp-up and ramp-down at the beginning and end of stimulation. We normalized the average MEP amplitudes for each post-stimulation time point to the average of 15 MEP amplitudes recorded before the intervention. Thus, changes in MEP amplitudes of each current direction were expressed as a ratio relative to their baseline response.

Differences in on-line and off-line performance of the skill measure were compared using a one-way analysis of variance (ANOVA). Multiple comparisons corrected post-hoc analysis was administered when appropriate. To evaluate the effects of tDCS on M1 excitability for each current direction (PA, AP), we used repeated-measures ANOVA with factors “MONTAGE” (Focal, Conventional, Sham) and “TIME” (P0, P15, P30). Here, post-stimulation MEP amplitudes normalized to baseline measures were used in the statistical analysis. When analysis showed no overall effect of TIME from P0-P30, we calculated the mean post-tDCS effect for post hoc testing. All data in text and figures are depicted as mean ± SEM.

To compare the amount of learning between the Focal, Conventional and Sham groups, we determined the impact of tDCS montage towards on-line (within Day 1) and off-line (between Day 2 start and Day 1 end) effects on the skill score (Fig. 1C). While we did not find any significant difference towards on-line effects between the three groups (F_[2,36]_ = 0.339, *p* = 0.716), one-way ANOVA revealed significant effects when considering off-line changes (F_[2,36]_ = 3.560, *p* = 0.042). We therefore conducted a *post-hoc* analysis for offline changes, which showed that only the Focal group was significantly different from the Sham group (*p* = 0.040), whereas no differences were found between the Focal and Conventional groups (*p* = 0.368) or Sham and Conventional groups (*p* > 0.90). Thus, these results indicate that only Focal tDCS improves overall motor skill retention in comparison to Sham stimulation.

To ensure that similar TMS intensities were applied between all groups, we conducted a one-way ANOVA with factor BASELINE RMT and found no differences between the three groups when considering PA-currents (F_[2,29]_ = 0.094, *p* > 0.9) and AP-currents (F_[2,29]_ = 0.036, *p* > 0.9). When we evaluated the effects of tDCS on M1 excitability evoked with PA-TMS currents, repeated-measures ANOVA demonstrated no effects of MONTAGE x TIME (F_[4,54]_ = 0.468, *p* = 0.759) or TIME (F_[2,54]_ = 1.234, *p* = 0.299), but did reveal a significant effect of MONTAGE (F_[2,54]_ = 5.002, *p* = 0.014; Fig. 2A). Collapsing all the post-tDCS PA-MEPs, we conducted a one-way ANOVA with factor MONTAGE and found differences across the experimental groups (F_[2,29]_ = 4.892, *p* = 0.015; Fig. 2A), with Bonferroni corrected post-hoc tests revealing that MEPs were significant larger for the Focal (*p* = 0.028) and Conventional (*p* = 0.045) when compared to Sham, but not between Focal and Conventional (*p* > 0.9, Fig. 2A). Similarly, when we evaluated the effects of tDCS on M1 excitability evoked with AP-TMS currents, repeated-measures ANOVA demonstrated no effects of MONTAGE x TIME (F_[4,54]_ = 1.386, *p* = 0.251) or TIME (F_[2,54]_ = 0.332, *p* = 0.719), but did reveal a significant effect of MONTAGE (F_[1,54]_ = 3.550, *p* = 0.043; Fig. 2B). Collapsing all the post-tDCS AP-MEPs, we conducted a one-way ANOVA with factor MONTAGE that revealed significant differences between groups (F_[2,29]_ = 3.546, *p* = 0.043). Bonferroni corrected post-hoc significant differences were found between Focal and Conventional (*p* = 0.040), but not between Focal and Sham (*p* = 0.906) or Conventional and Sham (*p* = 0.369).

We show that Focal tDCS led to greater motor skill performance in between-day retention but had no effect during training sessions. Enhanced retention was most prominent after the first training session, in which only the Focal group showed significant improvement when compared to Sham. While both montages showed facilitatory effects on the MEP responses measured with PA currents, only Focal tDCS showed inhibitory after-effects on MEP responses produced with AP currents when compared to conventional methods, indicating that Focal stimulation can bidirectionally modulate specific intracortical circuits that have unique synaptic input pathways to corticospinal neurons. Although preliminary, our findings suggest that Focal tDCS can enhance motor skill retention and may produce polarizing effects on cortico-excitability when applying directional TMS. One interpretation for the behavioral results with Focal tDCS is that limiting the stimulation over M1 and its surrounding regions is most important for enhancing motor learning. Whether this effect is due to differences in electrode placement or current density applied over M1 requires further study. While the effect sizes for the main analyses were moderate, the current results are limited by the sample size and by the matched, fixed dose of tDCS applied in both montages (Evans et al., 2020). Further studies with a larger sample size and individualized dose-control of tDCS are required to confirm the importance of focalized stimulation on motor learning.

## Funding

Work was supported by 10.13039/501100000265Medical Research Council (Grant MR/P006671/1)

## Declaration of Competing Interest

Authors declare no conflicts of interest.

## References

[bib0005] Ballard H.K., Eakin S.M., Maldonado T., Bernard J.A. (2021). Using high-definition transcranial direct current stimulation to investigate the role of the dorsolateral prefrontal cortex in explicit sequence learning. PLoS One.

[bib0010] Cantarero G., Spampinato D., Reis J., Ajagbe L., Thompson T., Kulkarni K., Celnik P. (2015). Cerebellar direct current stimulation enhances on-line motor skill acquisition through an effect on accuracy. J. Neurosci..

[bib0015] Cole L., Giuffre A., Ciechanski P., Carlson H.L., Zewdie E., Kuo H.C., Kirton A. (2018). Effects of high-definition and conventional transcranial direct-current stimulation on motor learning in children. Front. Neurosci..

[bib0020] Datta A., Bansal V., Diaz J., Patel J., Reato D., Bikson M. (2009). Gyri-precise head model of transcranial direct current stimulation: improved spatial focality using a ring electrode versus conventional rectangular pad. Brain Stimul..

[bib0025] Doppelmayr M., Pixa N.H., Steinberg F. (2016). Cerebellar, but not motor or parietal, high-density anodal transcranial direct current stimulation facilitates motor adaptation. J. Int. Neuropsychol. Soc..

[bib0030] Evans C., Bachmann C., Lee J., Gregoriou E., Ward N., Bestmann S. (2020). Dose-controlled tDCS reduces electric field intensity variability at a cortical target site. Brain Stimul..

[bib0035] Hamada M., Galea J.M., Di Lazzaro V., Mazzone P., Ziemann U., Rothwell J.C. (2014). Two distinct interneuron circuits in human motor cortex are linked to different subsets of physiological and behavioral plasticity. J. Neurosci..

[bib0040] Kuo H.I., Bikson M., Datta A., Minhas P., Paulus W., Kuo M.F., Nitsche M.A. (2013). Comparing cortical plasticity induced by conventional and high-definition 4 × 1 ring tDCS: a neurophysiological study. Brain Stimul..

[bib0045] Lefebvre S., Jann K., Schmiesing A., Ito K., Jog M., Schweighofer N., Wang D., Liew S.L. (2019). Differences in high-definition transcranial direct current stimulation over the motor hotspot versus the premotor cortex on motor network excitability. Sci. Rep..

[bib0050] Lerner O., Friedman J., Frenkel-Toledo S. (2021). The effect of high-definition transcranial direct current stimulation intensity on motor performance in healthy adults: a randomized controlled trial. J. Neuroeng. Rehabil..

[bib0055] Masina F., Arcara G., Galletti E., Cinque I., Gamberini L., Mapelli D. (2021). Neurophysiological and behavioural effects of conventional and high definition tDCS. Sci. Rep..

[bib0060] Rawji V., Ciocca M., Zacharia A., Soares D., Truong D., Bikson M., Rothwell J., Bestmann S. (2018). tDCS changes in motor excitability are specific to orientation of current flow. Brain Stimul..

[bib0065] Reis J., Schambra H.M., Cohen L.G., Buch E.R., Fritsch B., Zarahn E., Celnik P.A., Krakauer J.W. (2009). Noninvasive cortical stimulation enhances motor skill acquisition over multiple days through an effect on consolidation. Proc. Natl. Acad. Sci. U.S.A..

[bib0070] Rossi S., Hallett M., Rossini P.M., Pascual-Leone A., Safety of TMS Consensus Group (2009). Safety, ethical considerations, and application guidelines for the use of transcranial magnetic stimulation in clinical practice and research. Clin. Neurophysiol..

[bib0075] Spampinato D., Celnik P. (2021). Multiple motor learning processes in humans: defining their neurophysiological bases. Neuroscientist.

[bib0080] Wiethoff S., Hamada M., Rothwell J.C. (2014). Variability in response to transcranial direct current stimulation of the motor cortex. Brain Stimul..

[bib0085] Woods A.J., Antal A., Bikson M., Boggio P.S., Brunoni A.R., Celnik P., Cohen L.G., Fregni F., Herrmann C.S., Kappenman E.S., Knotkova H., Liebetanz D., Miniussi C., Miranda P.C., Paulus W., Priori A., Reato D., Stagg C., Wenderoth N., Nitsche M.A. (2016). A technical guide to tDCS, and related non-invasive brain stimulation tools. Clin. Neurophysiol..

